# Trust in societal factors and vaccine hesitancy in Western Balkans

**DOI:** 10.1093/eurpub/ckac130.156

**Published:** 2022-10-25

**Authors:** S Matovic-Miljanovic, S Cvjetkovic, V Jeremic-Stojkovic, S Mandic-Rajcevic, V Bjegovic-Mikanovic, M Gross

**Affiliations:** 1 Public Health, Euro Health Group, Copenhagen, Denmark; 2 Institute for Social Medicine, School of Medicine, University of Belgrade, Belgrade, Serbia; 3 Department of Humanities, School of Medicine, University of Belgrade, Belgrade, Serbia

## Abstract

**Background:**

Countries in the Western Balkans are facing vaccine hesitancy, trying to bring vaccine acceptance and ways to improve it into the focus. Trust in science and institutions, namely political trust, plays an important role and can significantly affects vaccine acceptance.

**Methods:**

Cross-sectional research was carried out from July to September 2021 in five countries of the Western Balkans (Albania, Bosnia and Herzegovina, North Macedonia, Montenegro and Serbia) and included adult population aged 18 and older (1605 individuals). Convenience sampling was applied using anonymised online questionnaires and Likert scales, shared through online social media, and asking, among others, for trust in societal factors.

**Results:**

In all countries people had more confidence in health authorities than in political officials. There are no gender differences found in showing trust in societal factors, except in Serbia where women compared to men showed greater trust in health authorities (50.6% vs. 34.4%), as well as in political officials (42.8% vs. 28.2%). The lowest trust in pharmaceutical companies was found in Albania where 34,9% respondents believe that vaccination against COVID-19 is largely promoted by pharmaceutical companies due to financial profits. People who put more trust in societal factors were vaccinated to a greater extent. In Serbia and Albania, the older respondents in general put more trust in societal factors. People who assessed themselves as more religious in Serbia and North Macedonia demonstrated less trust towards societal factors.

**Conclusions:**

The study demonstrated moderate trust in societal factors in all countries, with greatest trust in health authorities. This implies that health authorities should have a pivotal role, together with physicians in primary health, in promoting vaccination and educating the general public in the Western Balkans.

**Key messages:**

- Vaccine hesitancy is identified as one of the crucial contributors to the decline in vaccine coverage in the Western Balkans.
- Vaccine hesitancy is associated with the global crisis of trust in science and institutions.

